# Analysis of Residual Flexural Stiffness of Steel Fiber-Reinforced Concrete Beams with Steel Reinforcement

**DOI:** 10.3390/ma13122698

**Published:** 2020-06-13

**Authors:** Violetta K. Kytinou, Constantin E. Chalioris, Chris G. Karayannis

**Affiliations:** Laboratory of Reinforced Concrete and Seismic Design of Structures, Department of Civil Engineering, Faculty of Engineering, Democritus University of Thrace (D.U.Th.), 67100 Xanthi, Greece; vkytinou@civil.duth.gr (V.K.K.); karayan@civil.duth.gr (C.G.K.)

**Keywords:** reinforced concrete, steel fiber-reinforced concrete (SFRC), tension softening, tension stiffening, finite element (FE) analysis, smeared crack model, constitutive analysis, residual stresses, flexural behavior, numerical analysis

## Abstract

This paper investigates the ability of steel fibers to enhance the short-term behavior and flexural performance of realistic steel fiber-reinforced concrete (SFRC) structural members with steel reinforcing bars and stirrups using nonlinear 3D finite element (FE) analysis. Test results of 17 large-scale beam specimens tested under monotonic flexural four-point loading from the literature are used as an experimental database to validate the developed nonlinear 3D FE analysis and to study the contributions of steel fibers on the initial stiffness, strength, deformation capacity, cracking behavior, and residual stress. The examined SFRC beams include various ratios of longitudinal reinforcement (0.3%, 0.6%, and 1.0%) and steel fiber volume fractions (from 0.3 to 1.5%). The proposed FE analysis employs the nonlinearities of the materials with new and established constitutive relationships for the SFRC under compression and tension based on experimental data. Especially for the tensional response of SFRC, an efficient smeared crack approach is proposed that utilizes the fracture properties of the material utilizing special stress versus crack width relations with tension softening for the post-cracking SFRC tensile response instead of stress–strain laws. The post-cracking tensile behavior of the SFRC near the reinforcing bars is modeled by a tension stiffening model that considers the SFRC fracture properties, the steel fiber interaction in cracked concrete, and the bond behavior of steel bars. The model validation is carried out comparing the computed key overall and local responses and responses measured in the tests. Extensive comparisons between numerical and experimental results reveal that a reliable and computationally-efficient model captures well the key aspects of the response, such as the SFRC tension softening, the tension stiffening effect, the bending moment–curvature envelope, and the favorable contribution of the steel fibers on the residual response. The results of this study reveal the favorable influence of steel fibers on the flexural behavior, the cracking performance, and the post-cracking residual stress.

## 1. Introduction

The addition of randomly distributed discrete fibers in concrete significantly improves the overall performance of reinforced concrete (RC) structural members because of its enhanced tensile properties and cracking control. This improvement is mainly attributed to the crack-bridging phenomenon that is observed across crack surfaces due to the incorporated steel fibers. The advanced characteristics of fiber-reinforced cement-based elements depend on many factors such as size, type, elastic properties, aspect ratio, and volume fraction of fibers, and each type of fiber can be effective in some specific function [1,2].

Concrete can tolerate only a small tensile stress before it cracks in a quasi-brittle manner. Research into the use of short steel fibers as mass reinforcement in concrete over the last five decades or so has developed them as an option to mitigate the unfavorable cracking characteristics of concrete, due to the ability of the fibers to bridge and transmit tensile stresses across cracks [3,4]. Thus, cracking and, eventually, tensile failure of steel fiber-reinforced concrete (SFRC) elements requires debonding and eventually full pullout of the steel fibers crossing the developing crack. Consequently, stress situations that depend on the tensile strength of the material in the presence of steel fibers generally contribute toward the improvement of an energy-absorbing mechanism resulting in a substantially improved post-cracking behavior [5,6,7]. In this direction, the full or even partial replacement of traditional stirrups is crucial in shear-critical RC members because the use of SFRC reduces reinforcement congestion since in these cases design criteria require a high amount of transverse reinforcement that leads to a short spacing of stirrups [8,9,10,11,12].

Attempts have been made in the past to combine different types of fibers and their addition to cementitious composites to improve the cracking performance in concrete at different levels [13,14]. Small and soft fibers control the initiation and propagation of microcracks, whereas large and strong fibers control macrocracks. Such hybrid fiber-reinforced composites can also offer more attractive engineering properties because the presence of one type of fiber effectively uses the properties of the other fiber [15,16].

Tension stiffening is the ability of concrete to carry tension between cracks, which provides additional stiffness for a RC member in tension before the reinforcement yields [17]. It can significantly affect member rigidity, deflection, and width of cracks under service loads [18,19,20]. Increased tension stiffening was observed for the high-strength concrete specimens, and the higher reinforcement ratio decreases the tension stiffening effect [21]. The presence of steel fibers is effective in controlling splitting cracks and significantly increases the tension stiffening effect because SFRC can carry tensile stress through the crack [22,23,24,25,26].

Nevertheless, the rather limited utilization of SFRC in structural applications can be attributed to the difficulty in establishing reliable and rational design procedures that describe and predict the material’s behavior for each design limit state. At the strength limit state in flexure, a conservative view is often taken to ignore the tensile capacity of SFRC at critical design sections [27]. This approach is often defended by the fact that the fibers are randomly orientated within a concrete matrix as opposed to aligned in the direction oriented to the principal tensile stress [28]. However, as SFRC can carry tensile stresses not only at the cracks but also between them, as a result of bond, the member is stiffer because of the steel fibers. This phenomenon termed “tension stiffening” combines the fiber interaction in cracked concrete, the bond behavior of steel reinforcing bars, and fracture mechanics of concrete, and it needs to be considered in service calculations when designing structural members made of SFRC.

Further, it is known that, in most of the practical applications of SFRC construction, steel fibers are combined with conventional steel reinforcement such as transverse stirrups and longitudinal reinforcing bars. When both forms of reinforcement bridge a crack, the transmitted tensile stress across the crack is shared between the reinforcing bars and the fibers [29]. Consequently, the average steel strains are smaller than they would be without the fibers between the cracks, leading to more closely spaced cracks [30]. Thus, the addition of steel fibers effectively increases tension stiffening effect, improves crack control, and permits the use of higher strength reinforcing steels while still maintaining control of crack widths depending on the type and dosage of fibers used. Tension stiffening effects are also useful for assessing cracking behavior at service loads and to develop suitable material models of cracked SFRC for analysis that use averaged stresses and strains to predict member behavior [31,32,33].

In this paper, the effectiveness of steel fibers to improve the flexural response of realistic SFRC structural members with conventional steel reinforcement using nonlinear 3D finite element (FE) analysis is investigated. For this reason, an efficient FE model implemented in ABAQUS [34] software has been developed, considering the added steel fibers according to their type, aspect ratio, and percentage of the addition so that a more realistic calculation of the behavior of SFRC members is possible. The proposed model employs the nonlinearities of the materials by constitutive relationships for the SFRC under compression and tension based on experimental data in order to simulate the flexural performance of SFRC beams with steel reinforcing bars and stirrups accurately and reach results close to reality. Experimental testing is more expensive and time consuming compared to computer analysis, thus being able to produce reliable results by studying individual RC members and even structures by FE analysis is vital. Detailed and precise FE modeling can even replace experimental testing for research purposes and enable us to explore a much more extensive range of innovative design solutions and to solve complex engineering problems [35,36]. Seventeen analyses were executed using the proposed model, and the results are discussed in this paper. Comparing the FE models’ responses with the published experimental results reveals that the proposed model can successfully be used to predict the short-term behavior of SFRC beams under monotonic loading. Therefore, the favorable contribution of the steel fibers on the flexural behavior, the cracking performance, and the post-cracking residual strength and response are presented and discussed.

## 2. Constitutive Laws of the Materials and FE Model Formulation

The main aspects of the SFRC analyses developed in the current research are presented in this section. The constitutive laws of SFRC have been derived from experimental results and models proposed by the authors in previous studies available in the literature. Especially for the simulation of the SFRC under tension, a smeared crack approach is adopted. The proposed smeared crack model utilizes the fracture properties of the material and employs constitutive relationships of stress versus crack width with tension softening for the post-cracking SFRC tensile response instead of stress–strain laws. Further, the post-cracking tensile behavior of the SFRC near the reinforcing bars is modeled by a tension stiffening model that considers the SFRC fracture properties and the reinforcement characteristics.

### 2.1. Stages of SFRC Cracking and Tension Stiffening

**Figure 1:**
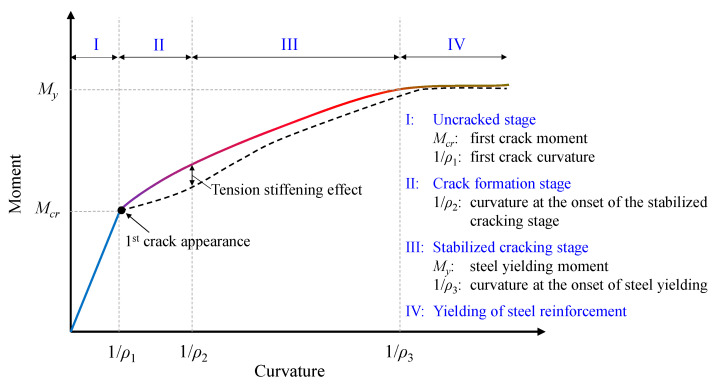
Stages of cracking and tension stiffening in SFRC members.

Figure 1 illustrates an idealized moment versus curvature response of a SFRC member including four stages of cracking. In the first stage (Stage I), concrete, as well as steel, are considered to behave in a linear and elastic way. Stresses and strains are uniformly distributed along the beam, and the applied load is shared between concrete and reinforcement. Concrete is considered macroscopically uncracked, and the tensile stress in concrete does not exceed its tensile strength. Steel fibers are assumed to not contribute at this stage, as a crack must be formed for the slip between concrete and fiber to occur, and fiber to be activated.

The second stage (Stage II) begins when the first crack is formed. Once tensile stress overcomes the tensile strength of SFRC and cracking moment is exceeded, a flexural crack begins to develop in the weakest cross-section of the beam. At this stage, the concrete–steel bond mechanism is activated, and the tension stiffening effect starts contributing to the member response. Tension stiffening is the ability of concrete between the cracks to bear tensile stresses, and through the bond mechanism, tensile stresses can be transferred from reinforcement to concrete. In addition, fibers tend to “bridge” the formed cracks, improve the bond efficiency due to the ability to also carry tension across cracks and thereby improve the tension stiffening performance of the member. As a result, the average tensile stiffness of cracked RC is greater than that of the reinforcing bars alone. Therefore, if this effect is disregarded, the stiffness of the cracked RC may be underestimated. As the imposed load increases, more cracks are developed, and the process goes on until the final pattern of cracking is established. If the space between the cracks is not large enough to cultivate a sufficient bond to allow the developed stress to reach tensile strength, no further cracking will occur.

The third stage (Stage III) occurs when the crack pattern has stabilized, and the crack spacing remains constant; cracks self-propagate at this point. The tension stiffening effect decays as the load increases. In the final stage (Stage IV), as the member’s average tensile stress begins to exceed the yield strain of the reinforcing bars, the member’s response is determined by the behavior of the fibers and the reinforcing bars.

### 2.2. Concrete Damaged Plasticity

The well-known concrete damaged plasticity (CDP) model is usually employed in FE analyses using Abaqus to simulate the nonlinear behavior of concrete. CDP is a general-purpose commonly used model that can be applied to all types of concrete structures under monotonic, cyclic, and dynamic loading. It is a continuum, plasticity-based, damage model that assumes tensile cracking and compressive crushing are the two main failure mechanisms in concrete [37]. In the FE analyses performed in this study, the CDP model has been used for comparison reasons to evaluate the effectiveness and the accuracy of the proposed materials model. Some main aspects and equations of the CDP model are briefly reported below.

The CDP model adopts the elastic model to define the mechanical properties of the materials in the elastic stage. However, when entering the stage of damage, to describe the stiffness degradation, the modulus of elasticity is described as:

(1) $$E=\left(1-d\right)E_{0}$$

where *E*_0_ is the initial elastic modulus and *d* is the plastic damage factor (*d_c_* or *d_t_*, for compression and tension, respectively), which varies from 0 ≤ *d* ≤ 1, with zero indicating the undamaged material, to one indicating a complete loss of strength.

According to Lubliner et al. [37], plastic degradation occurs only within the softening range and the stiffness is proportional to the material’s cohesion. Solving the previous equation for the plastic damage factor, it is derived:

(2) $$\frac{E}{E_{0}}=1-d=\frac{c}{c_{max}}\to d=1-\frac{c}{c_{max}}$$

where *c* is cohesion in the yield criteria, which is proportional to stress, and *c_max_* is proportional to the strength of the concrete.

Adopting Equation (2) to uniaxial tension or compression converts to the following expression:

(3) $$d=1-\frac{\sigma }{f}$$

where *f* is either the compressive or the tensile strength of concrete and σ is the compressive or the tensile stress, respectively.

**Figure 2:**
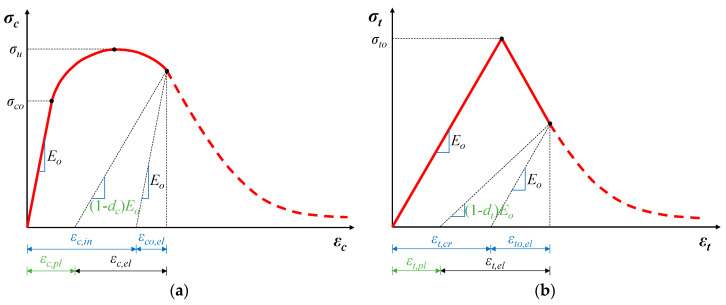
CDP model in Abaqus: (**a**) definition of compressive; and (**b**) definition of tensile damage.

The uniaxial compressive and tensile responses of concrete in relation to the concrete damage plasticity model subjected to compression and tension load are calculated based on the following expressions (see also Figure 2a,b for notation):

(4) $$\sigma_{c}=\left(1-d_{c}\right)E_{0}\left(\varepsilon_{c}-\varepsilon_{c,pl}\right)$$

(5) $$\sigma_{t}=\left(1-d_{t}\right)E_{0}\left(\varepsilon_{t}-\varepsilon_{t,pl}\right)$$

where $\sigma_{c}-\varepsilon_{c}$ and $\sigma_{t}-\varepsilon_{t}$ are the concrete compressive and tensile stress-strain relationships, respectively, $\varepsilon_{c,pl}$ and $\varepsilon_{t,pl}$ are the compressive and tensile plastic strain of concrete, respectively.

Using the above equations, the uniaxial stress–strain curves can be converted into stress versus plastic strain curves. This conversion is carried out by Abaqus automatically when user provides in a tabular form all the variables contained in the above equations. The strain variables for the compressive behavior are calculated as follows (see also Figure 2a for notation):

(6) $$\varepsilon_{c,in}=\varepsilon_{c,tot}-\varepsilon_{co,el},$$

(7) $$\varepsilon_{co,el}=\sigma_{co}/E_{0},$$

The equivalent plastic strain for crushed concrete, *ε_c,pl_*, is estimated using:

(8) $$\varepsilon_{c,pl}=\varepsilon_{c,in}-\frac{d_{c}}{1-d_{c}}\varepsilon_{co,el},$$

where *d_c_* is the compressive damage variable:

(9) $$d_{c}=1-\sigma_{c}/\sigma_{cu},$$

where and *σ_cu_* = *f_cm_* (mean concrete compressive strength).

In the same manner, the cracking strain ε*_t,cr_* and ε*_t_*_0*,el*_ for the tensile behavior are defined as (see also Figure 2b for notation):

(10) $$\varepsilon_{t,cr}=\varepsilon_{t,tot}-\varepsilon_{to,el},$$

(11) $$\varepsilon_{to,el}=\sigma_{to}/E_{0},$$

where the plastic strain for damaged concrete is:

(12) $$\varepsilon_{t,pl}=\varepsilon_{t,cr}-\frac{d_{t}}{1-d_{t}}\varepsilon_{to,el},$$

with *d_t_* the tensile damage variable:

(13) $$d_{t}=1-\sigma_{t}/\sigma_{to},$$

and *σ_to_* = *f_ctm_* (mean concrete tensile strength).

Five other CDP material associated parameters must be described to completely define the inelastic behavior of concrete [38,39,40]:

- *ψ* is the dilatation angle, which affects the amount of plastic volume deformation. Various values (20°–45°) are found in the literature. A dilation angle close to the friction angle of the material, for concrete 56.3°, results in a ductile behavior, whereas a low value near 0° leads to very brittle behavior.
- *∈*, the flow potential eccentricity, which is the rate of approach of the plastic potential hyperbolic to its asymptote, is used to define the shape of the plastic potential surface in the meridional plane.
- *σ_bo_/σ_co_* is the ratio of the strength in the biaxial state to the strength in the uniaxial state.
- *K_c_* is the ratio of the tensile to the compressive meridian and determines the shape of the yield surface.
- *μ* is the viscosity parameter, which is used to help the analysis achieve a good convergence.

The values used in the current study are presented in Table 1.

### 2.3. Proposed Model of the SFRC Compressive Behavior

Steel fibers practically become effective after cracking and consequently influence post-peak compressive behavior of SFRC. The amount, geometry, and the bond characteristics of the added steel fibers are the parameters affecting the improved post-cracking ductility of SFRC under compression. This enhancement is caused by the ability of steel fibers to exhibit a progressive debonding failure that provides crack-bridging, confinement, and crack growth resisting of the developed cracks [41,42,43,44]. However, compressive strength seems to be slightly increased, especially in SFRC mixtures with a low volume fraction of steel fibers. Most analytical models proposed for the simulation of the SFRC compressive response are based on regression analysis of experimental results available in the literature and taking into account the previously mentioned parameters [45,46,47,48,49,50].

**Figure 3:**
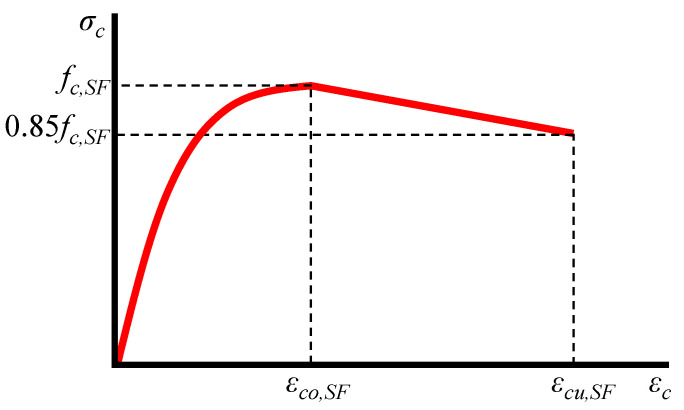
Stress–strain model of SFRC under compression.

The model adopted in this paper to simulate the compressive stress–strain (*σ_c_*–*ε_c_*) behavior of SFRC was proposed by Chalioris and Panagiotopoulos [51], and was derived from test data of 125 stress–strain curves and 257 strength values. Based on this model, the initial ascending compressive behavior until the maximum strength of common SFRC mixtures with cylinder compressive strength, *f_c,SF_*, less than 50 MPa is described by the expression (see also Figure 3 for notation):

(14) $$\sigma_{c}=f_{c,SF}\left[1-{\left(1-\frac{\varepsilon_{c}}{\varepsilon_{co,SF}}\right)}^{2}\right],$$

where *ε_co,SF_* is the SFRC strain that corresponds to the ultimate compressive stress.

The post-peak response is assumed to be linear from the value of the compressive strength, *f_c,SF_*, until the value of 0.85 *f_c,SF_*. The parameters of the strength, *f_c,SF_*, the strain at ultimate strength, *ε_co,SF_*, and the ultimate strain, *ε_cu,SF_*, that corresponds to the post-peak compressive stress 0.85 *f_c,SF_* are calculated using the following expressions:

(15) $$f_{c,SF}=f_{c}\left(0.2315F+1\right),$$

(16) $$\varepsilon_{co,SF}=\varepsilon_{co}\left(0.95F+1\right),$$

(17) $$\varepsilon_{cu,SF}=\varepsilon_{co,SF}\left(1.40F+1\right),$$

where *f_c_* is the cylinder compressive strength of plain concrete, *ε_co_* is the strain corresponding to the ultimate stress of plain concrete and usually takes the value of 0.002, and *F* is fiber factor that depends on the characteristics of the added steel fibers in the SFRC mixture and can be evaluated as: $F=\beta V_{SF}\left(l_{SF}/d_{SF}\right)$, where *β* is a bond factor that equals to 0.50 for round fibers, 0.75 for deformed (such as hooked, crimped, and undulated) fibers, and 1.0 for indented fibers, and *V_SF_*, *l_SF_*, and *d_SF_* are the volume fraction, length, the diameter of the steel fibers, respectively.

The ability of this analytical model to efficiently predict the overall compressive behavior of SFRC was verified against experimental stress–strain curves of various SFRC mixtures by Chalioris and Panagiotopoulos [51].

### 2.4. Proposed Smeared Crack Model of the SFRC Tensile Behavior

Steel fibers significantly improve the tensile performance of SFRC increasing the strength, mainly enhancing the deformation capability of the post-peak behavior by providing pseudo-ductile response due to their gradual debonding procedure [52]. Many analytical stress–strain expressions have been developed to describe the SFRC response under tension [53,54]. In this paper, a smeared crack analysis that has previously been developed and experimentally verified by the authors [55,56] to model the behavior of plain concrete under tension is properly modified and adopted for the SFRC tensile behavior. Similar smeared crack models have also been applied recently in FE simulations to evaluate the uncertainty of crack width of RC beams [57]. The adopted smeared crack model is properly modified herein to employ the favorable contribution of the added steel fibers on the tensile response.

**Figure 4:**
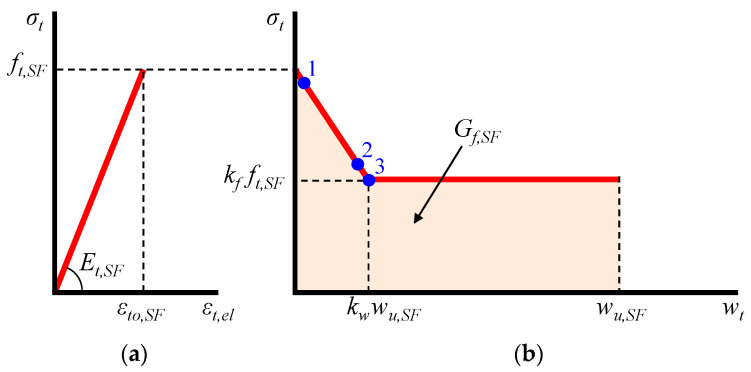
Tensile model of SFRC including: (**a**) stress versus strain elastic response; and (**b**) stress versus crack width post-cracking response with tension softening.

The proposed model employs constitutive relationships in terms of normal stress versus crack width for the post-cracking tensile response of SFRC instead of describing the crack process by stress–strain laws. Based on this aspect, crack propagation of SFRC takes place with the formation of a fracture process zone that is initiated at maximum tensile strength of SFRC, *f_t,SF_*, and is characterized by the gradually reduction of the strength during deformation. This fracture process zone is defined as the boundary of the strain softening region, which is considered as a SFRC property and is assumed to be wider than the region of visible cracks. It is also assumed that between the cracks of this zone there are less damaged or even elastic parts. Thus, the total tensional strain, *ε_t_*, is considered as the sum of an elastic, *ε_t,el_*, and a fracture component, *ε_t,fr_*, which can be estimated based on the following relationships (see also Figure 4):

(18) $$\varepsilon_{t}=\varepsilon_{t,el}+\varepsilon_{t,fr},$$

(19) $$\varepsilon_{t,el}=\sigma_{t}/E_{t,SF},$$

(20) $$\varepsilon_{t,fr}=w_{t}/L_{fr,SF},$$

where *σ_t_* is the tensile stress, *E_t,SF_* is the modulus of elasticity under tension, *w_t_* is the crack width, and *L_fr,SF_* is the length of fracture process zone of the SFRC.

It is noted that the influence of cracks on the deformations of the beams is considered through the implementation of the aforementioned smeared crack approach to the nonlinear FE analysis using Abaqus software to provide numerical behavioral curves and cracking patterns based on the stress distribution of the simulated SFRC beams.

#### 2.4.1. Elastic Response

The parameters of the elastic component of the proposed model for the simulation of the SFRC tensile behavior, as shown by the *σ_t_*–*ε_t,el_* linear diagram in Figure 4, are calculated based on the properties of the corresponding plain concrete and the added steel fibers, as shown in the following relationships [58]:

(21) $$f_{t,SF}=\varepsilon_{to,SF}E_{t,SF},$$

(22) $$\varepsilon_{to,SF}=0.167n_{l,el}V_{SF}\left(\varepsilon_{y,SF}-\varepsilon_{to}\right)+\varepsilon_{to},$$

(23) $$\varepsilon_{to}=f_{t}/E_{t},$$

(24) $$\varepsilon_{y,SF}=f_{SF}/E_{SF},$$

(25) $$E_{t,SF}=\frac{3}{8}\left[E_{t}\left(1-V_{SF}\right)+E_{SF}V_{SF}\right]+\frac{5}{8}\left[\frac{E_{SF}E_{t}}{E_{SF}\left(1-V_{SF}\right)+E_{t}V_{SF}}\right],$$

where *E_t,SF_* is the modulus of elasticity under tension of SFRC; *f_t_*, *ε_to_*, and *E_t_* are the ultimate tensile strength, the corresponding strain, and the modulus of elasticity under tension of the plain concrete, respectively; *f_SF_*, *ε_y,SF_*, and *E_SF_* are the ultimate tensile strength at yielding, the yield strain, and the modulus of elasticity of the steel fiber, respectively; and *n_l,el_* is the ratio of the average fiber elastic stress to the maximum fiber stress that is usually equal to 0.5.

#### 2.4.2. Fracture Response with Tension Softening

The fracture characteristics and the tension softening response of SFRC define the parameters of the fracture component of the proposed smeared crack model. The energy required for the formation of the cracks included in the fracture process zone and for the fully opening of one single crack for a unit area crack plane is the fracture energy, *G_f,SF_*, and can be expressed as:

(26) $$G_{f,SF}=\int_{f_{t,SF}}^{0}\sigma_{t}dw_{t}\overset{w_{t}=L_{fr,SF}\varepsilon_{t,fr}}{\to }G_{f,SF}=L_{fr,SF}\int_{f_{t,SF}}^{0}\sigma_{t}d.$$

The post-peak fracture response (*σ_t_*–*w_t_* curve in Figure 4) is described by a linear descending line until the point of the maximum post-cracking stress, *k_f_f_t,SF_*, and the corresponding crack width, *k_w_w_u,SF_*, and then a horizontal line of constant tensile stress (*σ_t_* = *k_t_f_t,SF_* when *w_t_* > *k_w_w_u,SF_*) until the ultimate considered crack width, *w_u,SF_*. Thus, the fracture energy can also be expressed in terms of the area under the curve of SFRC tensile stress versus crack width, as shown by the *σ_t_*–*w_t_* bilinear diagram in Figure 4:

(27) $$G_{f,SF}=k_{f}f_{t,SF}w_{u,SF}+0.5\left(f_{t,SF}-k_{\sigma }f_{t,SF}\right)k_{w}w_{u,SF}\to \;G_{f,SF}=f_{t,SF}w_{u,SF}\left(k_{f}+0.5k_{w}-0.5k_{f}k_{w}\right),$$

The values of the maximum post-cracking stress and crack width depend on the SFRC characteristics and can be estimated using the following expressions for short deformed steel fibers [58]:

(28) $$k_{f}f_{t,SF}=0.405n_{l}\sigma_{fu}V_{SF}\to k_{f}=\frac{0.405n_{l}\sigma_{fu}V_{SF}}{\varepsilon_{to,SF}E_{t,SF}}$$

(29) $$k_{w}w_{u,SF}=\left(3÷8\right)L_{fr,SF}\varepsilon_{to,SF}\to k_{w}=\frac{\left(3÷8\right)L_{fr,SF}\varepsilon_{to,SF}}{w_{u,SF}}$$

where *σ_fu_* is the ultimate stress of the fiber when a uniform ultimate bond stress, *τ_u_*, is assumed at the fiber–matrix interface and calculated as follows:

(30) $$\sigma_{fu}=\left\{\begin{array}{cc} 2\tau_{u}l_{SF}/d_{SF} & l_{SF}\leq l_{cr}\\ f_{SF} & l_{SF}>l_{cr} \end{array}\right\}$$

(31) $$n_{l}=\left\{\begin{array}{cc} 0.50 & l_{SF}\leq l_{cr}\\ 1-\frac{l_{SF}}{2l_{cr}} & l_{SF}>l_{cr} \end{array}\right\}$$

where *l_cr_* is the length in the fiber required to develop the ultimate fiber stress that can be estimated as:

(32) $$l_{cr}=0.5f_{SF}d_{SF}/\tau_{u}$$

#### 2.4.3. Fracture Energy

**Figure 5:**
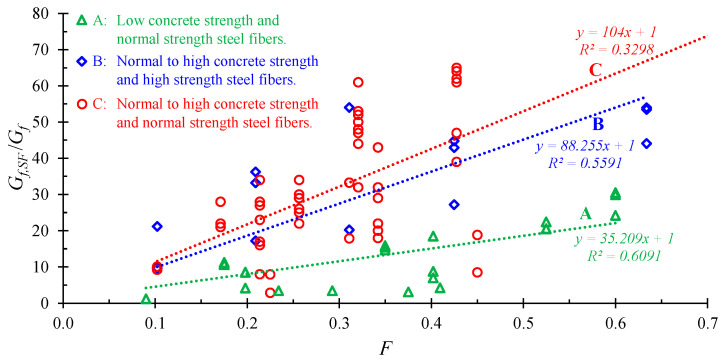
Estimation of SFRC fracture energy based on test results.

The value of the SFRC fracture energy is an important parameter that depends on the SFRC characteristics and can be evaluated experimentally by tension tests. In this study, the ratio of the fracture energy of the SFRC, *G_f,SF_*, to the fracture energy of the corresponding plain concrete, *G_f_*, was determined by 85 test results from the literature as a function of the fiber factor, *F*, as shown in Figure 5. The experimental results of low to high concrete strength deformed (hooked, crimped, and undulated) short steel fibers were used [59,60,61,62,63,64]. The experimental data points and fitted lines in Figure 5 include test results of low concrete strength with steel fibers of normal strength (Line A), normal to high concrete strength with steel fibers of high strength (Line B), and normal to high concrete strength with steel fibers of normal strength (Line C).

Thus, the expression derived from the experimental results presented in Figure 5 has the form:

(33) $$G_{f,SF}/G_{f}=k_{G}F+1\to G_{f,SF}=G_{f}\left(k_{G}F+1\right)$$

where *k_G_* is a factor that can be taken based on the results in Figure 5.

The fracture energy of the plain concrete, *G_f_*, can be expressed using the following relationship assuming a linear reduction of the tensile concrete stress from the maximum tensile strength, *f_t_*, to zero at the ultimate crack width, *w_u_*:

(34) $$G_{f}=0.5f_{t}w_{u}$$

(35) $$w_{u}=\varepsilon_{tu,fr}L_{fr}\overset{\varepsilon_{tu,fr}=a_{fr}\varepsilon_{to}}{\to }w_{u}=a_{fr}\varepsilon_{to}L_{fr}\overset{\left(23\right)}{\to }w_{u}=a_{fr}L_{fr}\frac{f_{t}}{E_{t}}$$

where *a_fr_* is a coefficient that depends on the shape of the stress versus crack width curve and the nature and size of the concrete aggregates and takes values from 5 to 8 for concrete aggregates of maximum size *d_g_* = 32 to 8 mm, respectively [55]. *L_fr_* is the length of fracture process zone of plain concrete that can be taken as 3*d_g_* [65].

Thus, using Equations (33)–(35), Equation (27) can be written as:

(36) $$f_{t,SF}w_{u,SF}\left(k_{f}+0.5k_{w}-0.5k_{f}k_{w}\right)=0.5f_{t}a_{fr}L_{fr}\frac{f_{t}}{E_{t}}\left(k_{G}F+1\right)\overset{L_{fr}=3d_{g}}{\to }$$

(37) $$w_{u,SF}=\frac{1.5f_{t}^{2}a_{fr}d_{g}\left(k_{G}F+1\right)}{f_{t,SF}E_{t}\left(k_{f}+0.5k_{w}-0.5k_{f}k_{w}\right)}$$

Based on the aspects of the proposed smeared crack model, the formulation of the stress is:

(38) $$\sigma_{t}=\left\{\begin{array}{cc} \varepsilon_{t}E_{t,SF} & \mathrm{if}\;0<\varepsilon_{t}\leq \varepsilon_{to,SF}\\ f_{t,SF}\left(1-\frac{1-k_{f}}{k_{w}w_{u,SF}}w_{t}\right) & \mathrm{if}\;0<w_{t}\leq k_{w}w_{u,SF}\\ k_{f}f_{t,SF} & \mathrm{if}\;k_{w}w_{u,SF}<w_{t}\leq w_{u,SF} \end{array}\right\}$$

#### 2.4.4. Fracture Process Zone of the SFRC

The aforementioned formulation can provide a feasible evaluation of the entire tensile behavior of the SFRC based on the elastic and fracture characteristics of the materials used in the fibrous mixture (concrete and steel fibers). All parameters and coefficients can be defined using the tensile strength, *f_t_*, the modulus of elasticity under tension, *E_t_*, and the maximum size of the aggregates, *d_g_*, of the plain concrete, along with the characteristics of the added steel fibers, such as their ultimate tensile strength at yielding, *f_SF_*, their modulus of elasticity, *E_SF_*, and the fiber factor, *F*, which depend on their characteristics (bond factor, *β*, volume fraction, *V_SF_*, length, *l_SF_*, and diameter, *d_SF_*). The only parameter that requires being defined is the size of the SFRC fracture process zone length, *L_fr,SF_*. This length cannot be assumed as mesh dependent, but it has to be attributed to the nature of the SFRC, the type of loading and the size of the specimen [55].

In this study the estimation of the crack process zone length is based on experimental data and the developed FE modeling. In the following sections, the methodology used to estimate the value of the fracture process zone length of the SFRC is presented.

### 2.5. Modeling of Steel Reinforcement

Steel reinforcement (longitudinal and stirrups) was modeled as a linear elastic material until the reach of yield point. Post-yielding behavior is considered to be nonlinear inelastic, referred as plastic behavior. Plastic behavior is characterized by permanent (plastic) deformations and is defined by introducing parameters of stress and plastic strain at the yield point (*f_y_*, *ε_y_*) as well as the stress and strain at ultimate point (*f_y_* and *ε_u_*). Steel modulus of elasticity, *E_s_*, is also provided to Abaqus, according to experimental data values as well as Poisson’s ratio, which is considered equal to 0.3 for all analyses performed in this study.

## 3. Application of the Proposed Model

### 3.1. Examined Beam Specimens

The current research presents a tension stiffening FE model for SFRC that properly considers the above-mentioned parameters. Seventeen SFRC beams available in the literature [66,67,68,69,70,71] were selected and a nonlinear FE analysis was performed to illustrate the capability and accuracy of the proposed model. The selected series of experimental tests had the same geometrical characteristics but differentiated in longitudinal reinforcement, concrete strength, and amount of steel fibers added to the concrete mixture.

These test series were chosen because all the necessary test data needed by the FE simulations were clearly reported. Further, the beam specimens include various ratios of longitudinal reinforcement (0.3%, 0.6%, and 1.0%) and steel fiber volume fractions (from 0.3 to 1.5%). Table 2 summarizes the geometric dimensions and reinforcement details of all examined beams as well as the material properties.

The 17 FE simulations were developed using same geometry, dimensions, material properties, and boundary conditions of the tested beams. The model is based on a 3D FE analysis implemented in Abaqus [34] software and on a smeared cracked approach, which does not require predetermined crack paths. Proper modeling and analysis of the SFRC beams involves full consideration on the material and geometry nonlinearities as well as effects related to the interaction and load sharing between steel and reinforced concrete. More details about the FE model are presented in the following sections.

### 3.2. Element Types Selected

**Figure 6:**
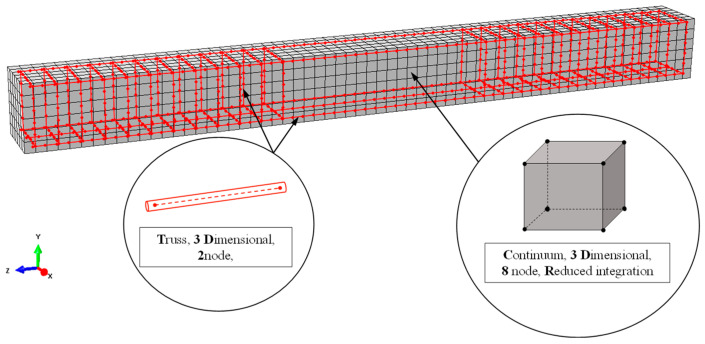
Concrete and steel reinforcement mesh.

SFRC was modeled using three-dimensional eight-node solid elements with reduced integration to prevent shear locking effect (C3D8R). For steel reinforcement (bars and stirrups), three-dimensional two-node truss elements were chosen (T3D2). Each node of these elements has three degrees of freedom with translation in X, Y, and Z directions (global coordinates system), as shown in Figure 6.

Proper modeling of steel reinforcement requires that bond interaction between reinforcement and concrete is considered. In general, the steel reinforcement could be modeled using a beam element with bending stiffness. However, in this study, the bending stiffness of the reinforcement is considered to be quite low compared to the bending stiffness of the SFRC matrix, and therefore it is neglected. Thus, truss elements are used for modeling steel reinforcement. The interaction between concrete and reinforcement is considered using the embedded method and specifically “the truss in solid” type. Truss elements (longitudinal reinforcement and stirrups) are embedded in solid region (SFRC), referred as host region. Geometric relationships between host and embedded elements are explored and if an embedded element node lies within a host element, then the transactional degrees of freedom (DOFs) of the node are constrained to follow the response of the host element.

### 3.3. Boundary Conditions and Loading

**Figure 7:**
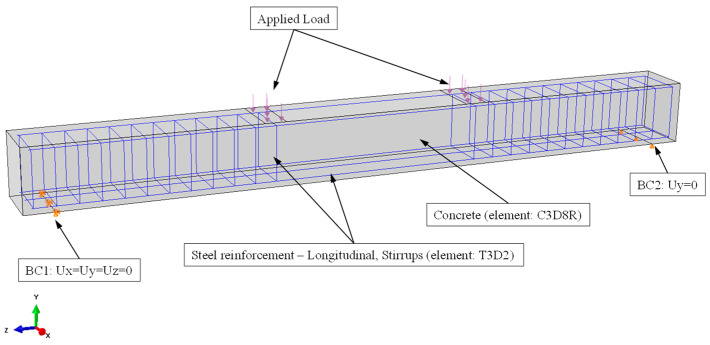
FE model boundary conditions and loading.

Displacement boundary conditions are necessary to constrain the model and obtain a unique solution. To ensure that the model behaves in an equivalent way as the experimental beam, the supports and loadings must be introduced to proper boundary conditions. The entire SFRC beam was modeled and simply supported. Two supports were designed in such a way as to create one roller and one pinned support. The supports were placed at a distance of 140 mm from each end of the beam according to the experimental set up, while the ends of the beams were free. At the one support, in the Ux, Uy, and Uz directions, a single line of nodes was constrained and added as constant values of 0, while at the other support only Uy direction were restricted given value of 0, as shown in Figure 7. This allows the beam to rotate at the supports.

The load was applied as a distributed surface load (Figure 7), which is more realistic as in real structures forces are most likely to be transferred through contact and thus a force is likely to be distributed across a contact area. Applying the load as a concentrated force to a single node can cause troublesome effects, such as high stress concentration around the application node. When a load is applied as surface load, the total force is distributed to the face of the FE which composes the surface, and then to the nodes laying on the face, which leads to having a smaller amount of the total force at each node and excessive local stress concentration is avoided. Further, to ensure that the dynamic implicit procedure adopted in this study obtained a quasi-static solution, the load was applied steadily and gradually to avoid any significant change in acceleration from one increment to the next. This further guarantees that the alterations in stresses and displacement are smooth. The loading sequence used in the FE analysis was based on the experimental sequence and the analysis was performed using the load control technique.

### 3.4. Meshing and Convergence

The mesh size of 50 mm was applied to all elements (truss and solid) to make sure that different materials (steel and concrete) share common nodes. This size of mesh was chosen because concrete cracking propagation usually involves spatial scales in the range of 2–3 dominant aggregate sizes (usually between 16 and 32 mm) of the concrete [65]. A convergence study was carried out using different mesh sizes before proceeding to the main analysis and the above-mentioned mesh proved to be fine enough to generate reliable results.

### 3.5. Material Input

The main properties of the proposed material models for each beam are presented in Table 2. Some additional parameters also used as input in the performed nonlinear analysis of the SFRC beams are presented in Table 3. Stress and crack widths at Points 1–3 and corresponding plastic damage factors shown in Table 3 are defined in the SFRC tension model depicted in Figure 4.

## 4. Results and Discussion

### 4.1. Validation and Accuracy of the Proposed Model

As mentioned in Section 2.4.4, the value of the SFRC fracture process zone length, *L_fr,SF_*, cannot be assumed as mesh dependent but it has to be attributed to the nature of the SFRC, the type of loading, and the size of the specimen. In this study the estimation of the proper length size for the specific loading case is based on the examined tests using the developed FE modeling and by the comparison of the numerical results and the experimental data.

**Figure 8:**
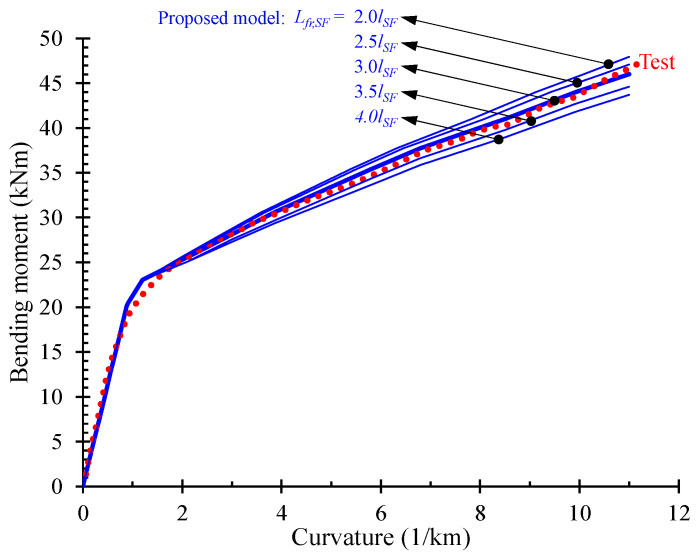
Evaluation of the SFRC fracture process zone length by comparisons between numerical and experimental bending moment versus curvature curves of beam S3-1-F05.

Thus, comparisons of the numerically predicted bending moment versus curvature responses of SFRC beams S3-1-F05 shown in Figure 8 for different values of fracture process zone length, *L_fr,SF_*, with the experimental response can yield useful conclusions about the proper size of this length and for this type of loading. The nature of the material used in this procedure is represented by the adopted stress versus crack width curve. The examined values of the fracture process zone length, *L_fr,SF_*, are taken as function of the steel fiber length, *l_SF_*, and five different values are investigated: *L_fr,SF_* = 2.0, 2.5, 3.0, 3.5 and 4.0 *l_SF_*. The comparisons of these five numerical curves (blue continuous lines) with the experimental data (red dotted line) shown in Figure 8 indicate that the best fitted numerical curve has been derived for *L_fr,SF_* = 3*l_SF_*. Further, the comparisons between the numerical predictions using this specific length of the SFRC fracture process zone the test results prove that the proposed model fits very well to the experimental behavior of the beams with higher accuracy than the simulation without considering the tension stiffening and softening effect.

**Figure 9:**
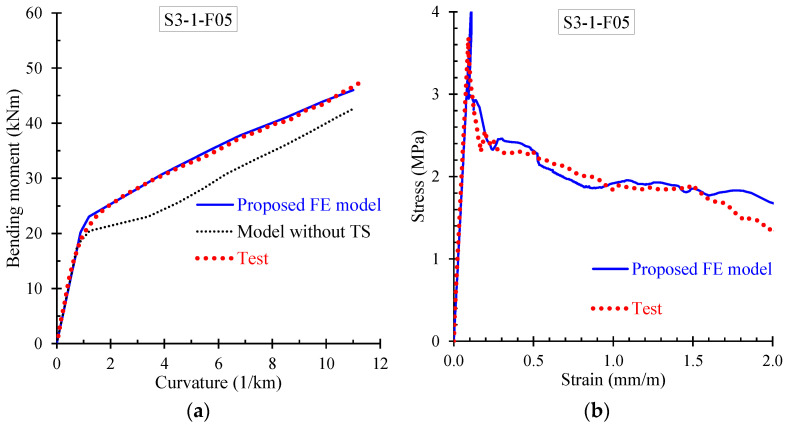
Comparisons between numerical curves and experimental results of beam S3-1-F05 in terms of: (**a**) bending moment versus curvature; and (**b**) residual stress versus strain curves.

**Figure 10:**
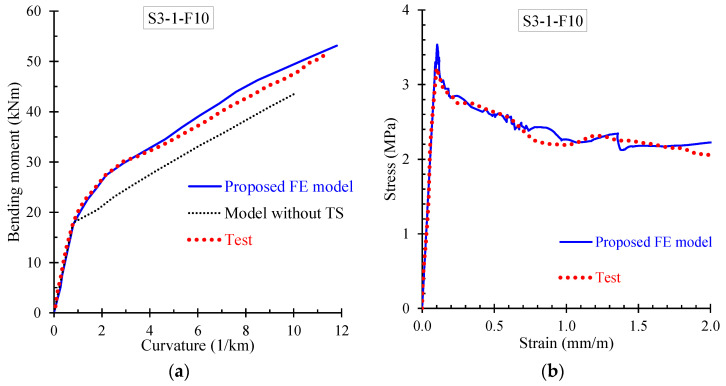
Comparisons between numerical curves and experimental results of beam S3-1-F10 in terms of: (**a**) bending moment versus curvature; and (**b**) residual stress versus strain curves.

**Figure 11:**
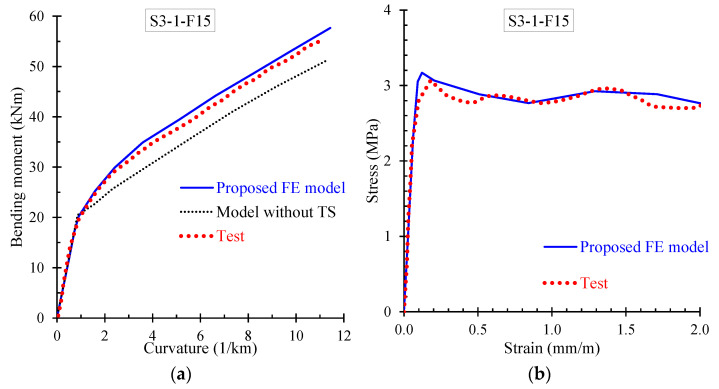
Comparisons between numerical curves and experimental results of beam S3-1-F15 in terms of: (**a**) bending moment versus curvature; and (**b**) residual stress versus strain.

**Figure 12:**
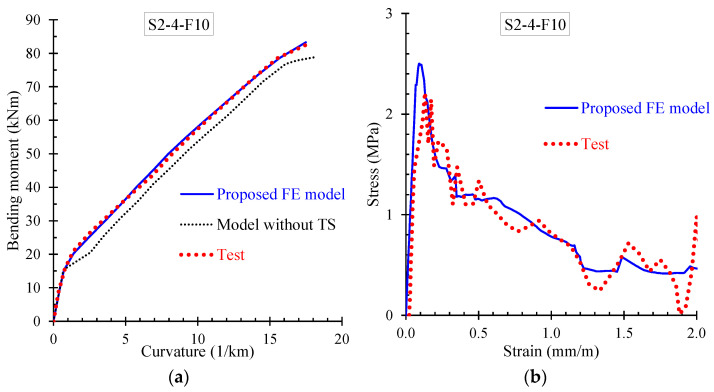
Comparisons between numerical curves and experimental results of beam S2-4-F10 in terms of: (**a**) bending moment versus curvature; and (**b**) residual stress versus strain.

Further, the numerical results derived from the proposed FE model are presented and compared with the corresponding experimental data in terms of: (a) bending moment versus curvature; and (b) stress versus strain curves. Especially, Figure 9, Figure 10, Figure 11 and Figure 12 demonstrate the FE analysis and the experimental behavioral curves of the SFRC beams S3-1-F05, S3-1-F10, S3-1-F15, and S2-4-F10 [67,68] with 0.5%, 1.0%, 1.5%, and 1.0% volume fraction of steel fibers, respectively. To comprehend the influence of the proposed smeared crack model with tension softening and the tension stiffening effect on the flexural behavior of the examined beams, two curves derived from FE analysis, in terms of bending moment versus curvature are compared with the experimental one in Figure 9a, Figure 10a, Figure 11a, and Figure 12a. The first curve (blue continuous line) was derived using the proposed material models in the nonlinear analysis with tension softening and tension stiffening approach and the second (black thin dotted line) using the FE model without considering these effects (denoted as “model without TS” where TS is tension softening and stiffening).

The presented stress versus strain curves in Figure 9b, Figure 10b, Figure 11b and Figure 12b consist of the stresses corresponding to the tension stiffening effect and the residual stresses due to fiber interaction with the concrete [67,68]. The numerical curves in these diagrams were calculated based on the stress–strain values in each element, which were extracted by the FE analysis and then the average values over all elements were calculated and used. The averaged stress strain values are the overall stress versus strain for the whole beam simulation and combined in a stress–strain curve.

It is noted that the point of the peak stress in the stress–strain diagrams of Figure 9b, Figure 10b, Figure 11b, and Figure 12b corresponds to the cracking point in the moment versus curvature diagram shown in Figure 9a, Figure 10a, Figure 11a, and Figure 12a, respectively. Since steel fibers mainly influence the post-cracking behavior of SFRC, the post-peak descending part of the residual stress versus strain curve depends on the volume fraction of the added steel fibers. Thus, SFRC mixtures with higher amount of fibers (for example, beam S3-1-F15 with *V_SF_* = 1.5%) exhibit higher post-cracking stress.

Further, the numerical predictions of the stress versus strain curves using the proposed nonlinear smeared crack model with tension softening and tension stiffening are in very good compliance with the experimentally measured values, as can clearly be observed for all examined beams in Figure 9b, Figure 10b, Figure 11b and Figure 12b.

**Figure 13:**
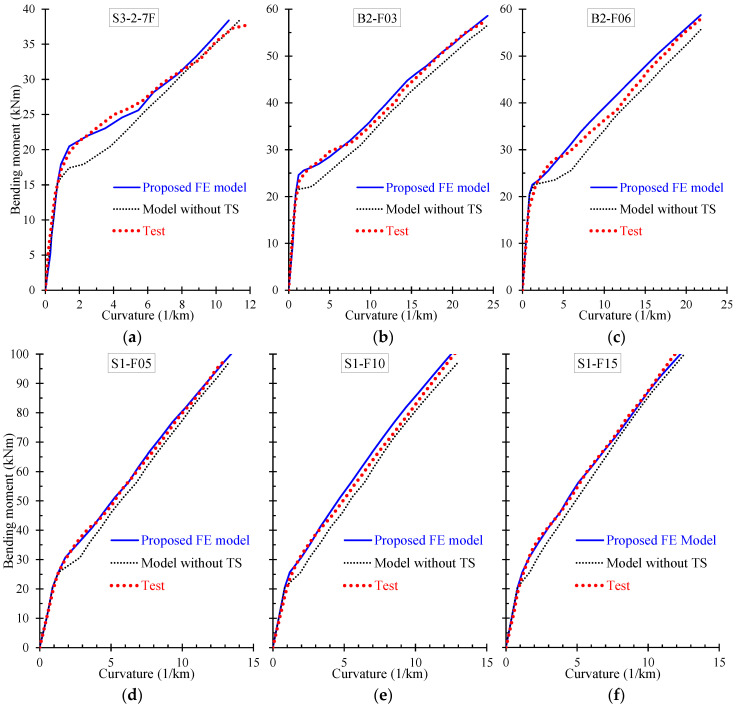
Comparisons between bending moment versus curvature numerical and experimental curves (model without TS: without tension softening and stiffening effect): (**a**) S3-2-7F; (**b**) B2-F03; (**c**) B3-F06; (**d**) S1-F05; (**e**) S1-F10; and (**f**) S1-F15.

Numerical curves derived by the developed nonlinear FE model with and without tension softening and stiffening effect are also compared with the experimental ones in the diagrams of Figure 13 for the SFRC beams: (a) S3-2-7F; (b) B2-F03; (c) B3-F06; (d) S1-F05; (e) S1-F10; and (f) S1-F15 [66,70,71]. These comparisons reveal that in most of the examined cases the numerical predictions of the proposed FE model that considers the smeared crack analysis with tension softening and tension stiffening effect are in closer agreement with the test data than the numerical curves without this effect.

**Figure 14:**
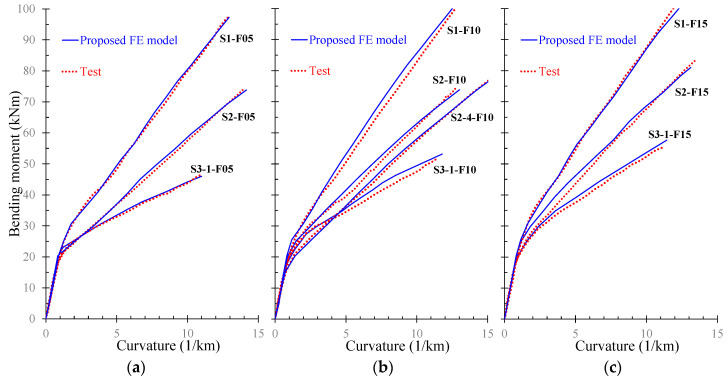
Numerical and test curves of SFRC with different steel fiber and longitudinal reinforcement ratios: (**a**) beams with *V_SF_* = 0.5%; (**b**) beams with *V_SF_* = 1.0%; and (**c**) beams with *V_SF_* = 1.5%.

The effectiveness of the proposed nonlinear FE model to predict accurately the flexural response of SFRC beams with different longitudinal reinforcement ratios is examined in the diagrams of Figure 14 in terms of bending moment versus curvature behavioral curves. The SFRC beams compared in the diagrams of Figure 14a–c has steel fiber volume fraction 0.5%, 1.0%, and 1.5%, respectively. Further, the ratio of the steel longitudinal reinforcement of the beams are as follows:

- Beams of series “S1” (S1-F05, S1-F10 and S1-F15): *ρ_l_* = 1.0%.
- Beams of series “S2” (S2-F05, S2-F10, S2-4-F10 and S2-F15): *ρ_l_* = 0.6%.
- Beams of series “S3” (S3-1-F05, S3-1-F10 and S3-1-F15): *ρ_l_* = 0.3%.

The comparisons shown in Figure 14 reveal that a good agreement between the numerical and the experimental bending moment versus curvature curves was achieved.

Further, to establish the validity of the proposed nonlinear FE analysis, Table 4 presents the calculated values of the differences between the numerical predictions and the test results in terms of “calculation errors”. Specifically, the discrepancy of the bending moment values between the numerical predictions and the tests for the same curvatures along the entire bending moment versus curvature diagrams of each examined beam was calculated to validate, statistically compare, and estimate the overall accuracy of the proposed model.

This discrepancy in terms of error percentage for each point was calculated according to the following known expression:

(39) $$Error\;\left(\%\right)=\left|\frac{M_{FE\;model}-M_{exp}}{M_{exp}}\right|\times 100$$

where *M_FE model_* and *M_exp_* are the bending moment values derived from the proposed FE model and the experiments, respectively.

Table 4 presents the values of the mean absolute error (MAE), the standard error (SE), and coefficient of variation (CV). These values indicate that model predictions lead to an error below 9% for every SFRC beam examined and the average value of MAE of all beams is 5.1%. 

### 4.2. Influence of Steel Fibers on the Flexural Performance

**Figure 15:**
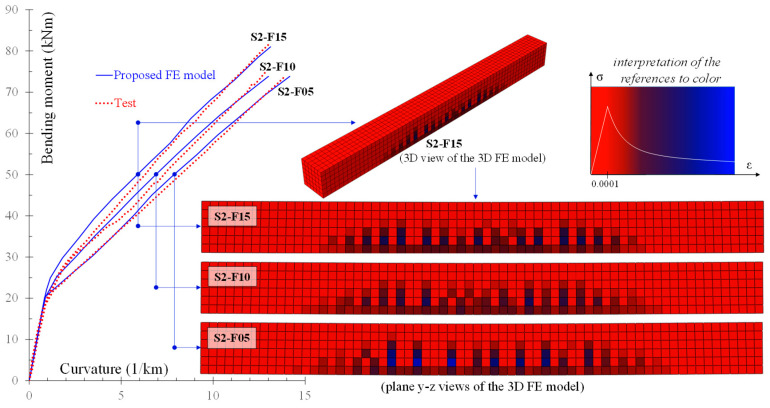
Influence of steel fibers on the bending moment versus curvature curves and the cracking pattern–stress distribution (plane y-z view of 3D model).

Figure 15 includes a diagram with the experimental and the numerical behavioral curves of beams S2-F05, S2-F10, and S2-F15 [69]. In the same figure, the schemes of the cracking pattern and the stress distribution of the beams derived by the proposed model at bending moment 50 kNm are also compared. For simplicity and comparison reasons, these numerical schemes show the plane y-z view of the 3D FE model of the beams and only beam S2-F15 is illustrated in both 3D and plane views. It is noted that SFRC beam S2-F15 with higher amount of steel fibers (*V_SF_* = 1.5%) demonstrates higher strength and lower deformation than the other SFRC beams with 1.0% and 0.5% steel fiber volume fraction. Further, this SFRC beam (specimen S2-F15) exhibits increased number of cracks and decreased crack spacing and width with respect to the cracking performance of beam S2-F05 with lower amount of steel fibers (*V_SF_* = 0.5%) due to the favorable contribution of the steel fibers. These observations are based on the numerical cracking pattern and stress distribution derived from the proposed FE analysis (Figure 15). The same observations have also been established by relevant tests of flexural SFRC beams [72,73,74].

**Figure 16:**
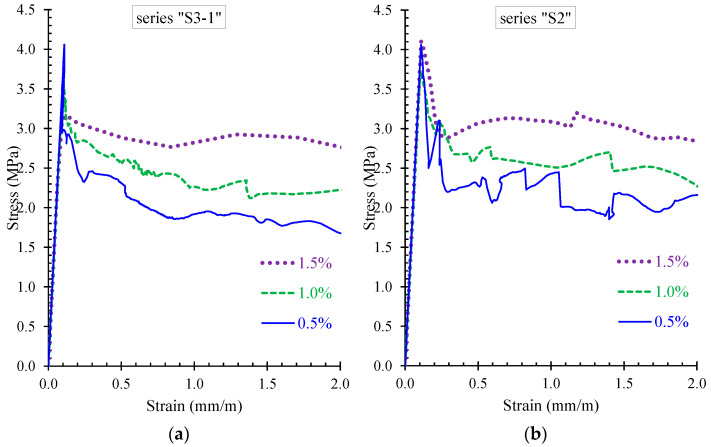
Influence of steel fibers on the residual strength of beams of series: (**a**) “S3-1”; and (**b**) “S2”.

Further, the contribution of steel fibers on the increase of the residual strength and the overall post-cracking stress versus strain behavior of SFRC structural members with steel bars and stirrups is demonstrated in Figure 16. Figure 16a presents the residual stress versus strain response derived from the proposed model of beams S3-1-F05, S3-1-F10, and S3-1-F15 with *V_SF_* = 0.5%, 1.0%, and 1.5% (beams of series “S3-1”) and Figure 16b presents the corresponding numerical response of beams S2-F05, S2-F10, and S2-F15 with *V_SF_* = 0.5%, 1.0%, and 1.5% (beams of series “S2”). In both examined cases, it is indicated that the increase of the volume fraction of the added steel fibers causes a gradual increase of the residual strength.

## 5. Conclusions

The influence of steel fibers on the short-term flexural behavior of realistic SFRC structural members reinforced with steel reinforcement was investigated by nonlinear 3D finite element (FE) analysis using software Abaqus. A database of 17 large-scale beam specimens collected from the literature was used as experimental baseline to perform the developed analysis that considers the tension stiffening effect and the nonlinearities of the materials by new and established constitutive relationships for steel fiber-reinforced concrete (SFRC) under compression and tension. The appropriate loading and constraint conditions were also considered in order for the performed FE analyses to be very close to experimental conditions. Based on the results of this study, the following conclusions can be drawn:

- The proposed FE model considers the contribution of the steel fibers according to their type, aspect ratio, and volume fraction to achieve a more realistic evaluation of the compressive and tensile behavior of SFRC utilizing well-established constitutive laws based on experimental data. Especially, for the simulation of the SFRC under tension a smeared crack approach is proposed that utilizes the fracture properties of the material and employs constitutive relationships of stress versus crack width with tension softening for the post-cracking SFRC tensile response instead of stress–strain laws. The post-cracking tensile behavior of the SFRC near the reinforcing bars is modeled by a tension stiffening model that considers the SFRC fracture properties and the reinforcement characteristics.
- The size of the fracture process zone of the SFRC under tension is attributed to the material, the type of loading, and the size of the specimen. An estimation of this length based on comparisons between numerical and experimental data is presented.
- The examined realistic SFRC structural members with increased values of the fiber factor, *F*, exhibit enhanced flexural performance in terms of initial stiffness, strength, deformation capacity, cracking behavior, and residual stress. The developed FE analysis predicts accurately the overall experimental flexural behavior and points out the contribution of the steel fibers on this improvement.
- Beams with higher amount of steel fibers (*V_SF_* = 1.5%) demonstrated lower deformation than the other SFRC beams with 1.0% and 0.5% steel fiber volume fraction at the same level of the applied load. Further, the favorable contribution of the added steel fibers on the cracking performance of the examined SFRC beams is also highlighted in the numerical results of this study. Beams with 1.5% steel fiber volume fraction showed increased number of cracks and decreased crack spacing and width with respect to the corresponding beams with lower amount of fibers (*V_SF_* = 0.5%).
- The favorable contribution of the added steel fibers on the post-cracking short-term behavior of realistic SFRC structural members with reinforcing bars and stirrups was revealed by the performed analyses. Numerical results of the examined SFRC beams show that the post-peak descending part of the residual stress versus strain curves depends on the volume fraction of the added steel fibers and SFRC mixtures with higher amounts of fibers exhibit higher post-cracking stress.
- Comparisons between experimental results and numerical predictions include typical bending moment versus curvature behavioral curves, deformation–cracking patterns, and residual stress versus strain diagrams. The proposed nonlinear smeared crack analysis with tension softening and tension stiffening effect was found to be reliable and capable of accurately simulating the flexural response of large-scale SFRC beams with steel reinforcement since the predicted behavioral curves are in good agreement with the corresponding experimental ones.

## Figures and Tables

**Table 1:** Parameters input of the CDP model.

Parameter	Value
*ψ*	31°
*K_c_*	2/3
*σ_b_*_0_ */σ_c_*_0_	1.16
*∈*	0.10
*μ*	0.0001

**Table 2:** Characteristics of the examined beams.

Ref.	Beam Name	Geometry		Long. Reinforcement			Material Properties			Steel Fiber Properties *				SFRC Properties (Model)					
		*b* (mm)	*h* (mm)	*A_s_*_1_ (mm^2^)	*A_s_*_2_ (mm^2^)	*ρ_l_* (%)	*f_cm_* (MPa)	*E_s_* (GPa)	*f_y_* (MPa)	*V_SF_* (%)	$\frac{l_{SF}}{d_{SF}}$	*F*	*f_SF_* (MPa)	*f_c,SF_* (MPa)	*ε_cu,SF_* (mm/m)	*f_t,SF_* (MPa)	*k_f_*	*ε_to,SF_* (mm/m)	*k_w_*
[66]	S3-2-7F	284	298	232 (3Ø10)	56 (2Ø6)	0.3	44.6	203	560	0.3	55/1	0.12	1020	45.88	2.98	3.37	0.18	0.097	0.56
[67]	S3-2-3	284	298	232 (3Ø10)	57 (2Ø6)	0.3	50.9	210	578	-	-	-	-	-	-	-	-	-	-
	S3-1-F05	278	302	235 (3Ø10)	56 (2Ø6)	0.3	55.6	203	560	0.5	53/1	0.20	1020	58.06	3.70	4.06	0.24	0.109	0.21
	S3-1-F10	279	300	235 (3Ø10)	56 (2Ø6)	0.3	48.0	203	560	1.0	53/1	0.40	1020	52.42	4.98	3.74	0.40	0.104	0.12
	S3-1-F15	279	300	235 (3Ø10)	56 (2Ø6)	0.3	52.2	203	560	1.5	53/1	0.60	1020	59.13	6.86	4.10	0.67	0.110	0.14
[68]	S2-4-F10	270	301	452 (4Ø12)	57 (2Ø6)	0.6	38.4	211	632	1.0	55/1	0.41	1020	42.07	4.76	3.13	0.36	0.093	0.08
[69]	S2-3	272	300	466 (3Ø14)	57 (2Ø6)	0.6	48.1	211	632	-	-	-	-	-	-	-	-	-	-
	S2-F05	273	301	477 (3Ø14)	56 (2Ø6)	0.6	55.6	205	559	0.5	53/1	0.20	1020	58.06	3.70	4.06	0.24	0.109	0.16
	S2-F10	272	301	477 (3Ø14)	56 (2Ø6)	0.6	48.0	205	559	1.0	53/1	0.40	1020	52.42	4.98	3.74	0.46	0.104	0.14
	S2-F15	272	299	477 (3Ø14)	56 (2Ø6)	0.6	52.2	205	559	1.5	53/1	0.60	1020	59.13	6.86	4.10	0.60	0.110	0.13
[70]	B1-F0	280	300	226 (2Ø12)	56 (2Ø6)	0.3	64.5	194	800	-	-	-	-	-	-	-	-	-	-
	B2-F03	280	300	226 (2Ø12)	56 (2Ø6)	0.3	65.3	194	800	0.3	50/1	0.11	1100	66.93	3.28	4.36	0.13	0.112	0.27
	B3-F06	280	300	226 (2Ø12)	56 (2Ø6)	0.3	62.3	194	800	0.6	50/1	0.23	1100	65.42	4.02	4.35	0.22	0.113	0.08
[71]	S1-3	280	300	755 (3Ø18)	57 (2Ø6)	1.0	48.1	211	632	-	-	-	-	-	-	-	-	-	-
	S1-F05	280	300	798 (3Ø18)	56 (2Ø6)	1.0	55.6	205	559	0.5	53/1	0.20	1020	58.06	3.70	4.06	0.24	0.109	0.25
	S1-F10	280	300	798 (3Ø18)	56 (2Ø6)	1.0	48.0	205	559	1.0	53/1	0.41	1020	52.58	5.10	3.74	0.42	0.104	0.13
	S1-F15	280	300	798 (3Ø18)	56 (2Ø6)	1.0	52.2	205	559	1.5	53/1	0.60	1020	59.13	6.86	4.10	0.60	0.110	0.12

* Hooked steel fibers.

**Table 3:** Input parameters of the examined SFRC beams.

Ref.	Beam Name	*E_t,SF_ = Ε_o_* (GPa)	*ν*	*G_f,SF_* (N/mm)	*σ_t_*_1_ (MPa)	*w_t_*_1_ (mm)	*ε_t_*_1_ (‰)	*σ_t_*_2_ (MPa)	*w_t_*_2_ (mm)	*ε_t_*_2_ (‰)	*σ_t_*_3_ (MPa)	*w_t_*_3_ (mm)	*ε_t_*_3_ (‰)	*d_t1_*	*d_t2_*	*d_t3_*
[66]	S3-2-7F	34.70	0.205	0.277	3.11	0.00990	0.06	0.81	0.10065	0.61	0.60	0.15675	0.95	0.076	0.761	0.822
[67]	S3-1-F05	37.23	0.207	0.534	3.63	0.01113	0.07	1.05	0.08109	0.51	0.97	0.16854	1.06	0.106	0.741	0.762
	S3-1-F10	36.06	0.215	0.893	3.32	0.01113	0.07	1.67	0.05565	0.35	1.50	0.15741	0.99	0.110	0.553	0.597
	S3-1-F15	37.38	0.222	1.394	3.85	0.01113	0.07	2.84	0.05406	0.34	2.74	0.16218	1.02	0.062	0.308	0.333
[68]	S2-4-F10	33.78	0.215	0.671	2.57	0.01155	0.07	1.45	0.03300	0.20	1.11	0.14685	0.89	0.179	0.537	0.644
[69]	S2-F05	37.23	0.207	0.534	3.48	0.01272	0.08	1.20	0.06042	0.38	0.97	0.16854	1.06	0.141	0.706	0.762
	S2-F10	36.06	0.215	0.893	3.36	0.01113	0.07	1.87	0.05406	0.34	1.72	0.15741	0.99	0.100	0.500	0.540
	S2-F15	37.38	0.222	1.394	3.80	0.01113	0.07	2.57	0.05565	0.35	2.45	0.16377	1.03	0.075	0.374	0.404
[70]	B2-F03	38.88	0.204	0.393	3.93	0.01050	0.07	0.97	0.08850	0.59	0.55	0.16650	1.11	0.097	0.777	0.875
	B3-F06	38.59	0.208	0.687	3.41	0.01350	0.09	1.54	0.03900	0.26	0.97	0.16500	1.10	0.216	0.647	0.776
[71]	S1-F05	37.23	0.207	0.564	3.72	0.01113	0.07	1.31	0.08904	0.56	0.97	0.16854	1.06	0.085	0.677	0.762
	S1-F10	36.06	0.215	0.876	3.33	0.01113	0.07	1.72	0.05406	0.34	1.56	0.15741	0.99	0.107	0.549	0.583
	S1-F15	37.38	0.222	1.430	3.80	0.01113	0.07	2.57	0.05565	0.35	2.45	0.16377	1.03	0.075	0.374	0.404

**Table 4:** Error calculation.

Ref.	Beam Name	*V_SF_* (%)	MAE	SE	CV
[66]	S3-2-7F	0.29	3.8%	0.9%	23%
[67]	S3-1-F05	0.5	5.0%	1.1%	22%
	S3-1-F10	1.0	6.1%	0.9%	15%
	S3-1-F15	1.5	7.3%	1.4%	19%
[68]	S2-4-F10	1.0	4.5%	0.9%	19%
[69]	S2-F05	0.5	4.6%	0.7%	14%
	S2-F10	1.0	4.2%	0.7%	17%
	S2-F15	1.5	8.7%	1.1%	13%
[70]	B2-F03	0.3	5.2%	1.4%	27%
	B3-F06	0.6	5.7%	0.8%	14%
[71]	S1-F05	0.5	2.3%	0.5%	22%
	S1-F10	1.0	5.2%	1.1%	21%
	S1-F15	1.5	3.2%	1.2%	36%
	average:		5.1%	1.0%	20%
